# Bergenin as a Novel Urate-Lowering Therapeutic Strategy for Hyperuricemia

**DOI:** 10.3389/fcell.2020.00703

**Published:** 2020-07-29

**Authors:** Mo Chen, Chenyi Ye, Jianing Zhu, Peiyu Zhang, Yujie Jiang, Xiaoyong Lu, Huaxiang Wu

**Affiliations:** ^1^Department of Rheumatology, The Second Affiliated Hospital, School of Medicine, Zhejiang University, Hangzhou, China; ^2^Department of Orthopedic, The Second Affiliated Hospital, School of Medicine, Zhejiang University, Hangzhou, China

**Keywords:** hyperuricemia, bergenin, ABCG2, SLC2A9, urate-lowering therapeutic

## Abstract

Bergenin is a C-glucoside of 4-*O*-methyl gallic acid isolated from several medicinal plants and has multiple biological activities. The aim of this study was to assess the potential usefulness of bergenin in hyperuricemia. We found that bergenin reduced serum urate levels in hyperuricemia mice by promoting renal and gut uric acid excretion. Bergenin treatment increased *Abcg2* expression both in the kidneys and intestine, while the expression of *Slc2a9* was suppressed in the kidney and increased in the intestine. Moreover, bergenin induced *ABCG2* expression in HK-2 and Caco-2 cells, as well as *SLC2A9* in Caco-2 cells, via the activation of *PPAR*γ. Nevertheless, bergenin suppressed *SLC2A9* expression in HK-2 cells by inhibiting the nuclear translocation of p53. Furthermore, bergenin decreased the serum levels of IL-6, IL-1β, and TNF-α in hyperuricemia mice, and promoted a polarization shift from the M1 to M2 phenotype in RAW264.7 cells. In conclusion, these findings provide evidence supporting the further development of bergenin as a novel therapeutic strategy for hyperuricemia.

## Introduction

The global burden of gout remains substantial, and in many parts of the world, its incidence has increased over the past years (Kuo et al., 2015). In 2015–2016, the prevalence of hyperuricemia in the United States was 20.2% (22.8 million) in males and 20.0% (24.4 million) in females (Chen-Xu et al., 2019). Moreover, individuals with asymptomatic hyperuricemia are at high risk of developing a variety of diseases, including gouty arthritis, renal damage, hypertension, diabetes mellitus, and metabolic syndrome (Dalbeth et al., 2019). Recent evidence has shown a link between high urate exposure and an increased inflammatory capacity across several tissues and immune cell types (Joosten et al., 2020). Urate-lowering therapies (ULT), including xanthine oxidase inhibitors and uricosuric drugs, often cause severe side effects. Therefore, according to evidence-based international guidelines, such therapies are only recommended in people with established conditions, such as gout and kidney stones (Dalbeth et al., 2019; Joosten et al., 2020). Hence, the development of safe and effective hyperuricemia therapies remains an unmet clinical need.

Nowadays it has become clearly that altered urate transport, both in the gut and the kidneys, has a central role in the pathogenesis of hyperuricaemia and gout (Dalbeth et al., 2019). Furthermore, genome-wide association studies (GWAS) have identified numerous loci that are associated with hyperuricemia and gout (Kawamura et al., 2019; Nakatochi et al., 2019). Serum urate levels are primarily regulated by the activity of the four transporters solute carrier family 2, facilitated glucose transporter member (*SLC2A9*), solute carrier family 22 member 12(*SLC22A12*), solute carrier family 17 member 1 (*SLC17A1*), and ATP-binding cassette transporter, subfamily G, member 2 (*ABCG2*), in the kidneys, and of *ABCG2* in the intestine (Nakayama et al., 2017).

Bergenin is the C-glucoside of 4-*O*-methyl gallic acid and can be found in several medicinal plants, including *Bergenia crassifolia* and *Corylopsis spicata* (Liang et al., 2017). Bergenin has been reported to have multiple biological activities, including antiarthritic (Jain et al., 2014; Singh et al., 2017), immunomodulatory (Wang et al., 2017; Kumar et al., 2019), antidiabetic (Veerapur et al., 2012), osteogenic (Hou et al., 2019), neuroprotective (Barai et al., 2019; Ji et al., 2019), and wound-healing effects (Mukherjee et al., 2013). Wang et al. (2017) showed that bergenin attenuated colitis by activating the peroxisome proliferator-activated receptor (PPAR) γ. Moreover, Veerapur et al. (2012) found that bergenin exerted the antidiabetic effect and could bind the PPAR ligand-binding domain. *PPAR*γ regulates the expression of various genes by directly binding to peroxisome proliferator response elements (Mandard and Patsouris, 2013).

Although *PPAR*γ has been reported to regulate the expression of *ABCG2* (Szatmari et al., 2006; To and Tomlinson, 2013; Wang et al., 2016), the relevance of bergenin as a therapeutic agent for hyperuricemia remains unclear. The aim of this study was to explore the potential clinical usefulness of bergenin in hyperuricemia. To this end, we investigated the effects of bergenin on hyperuricemia *in vitro* and *in vivo*, as well as the potential biological mechanisms underlying these effects.

## Materials and Methods

### Reagents and Antibodies

Bergenin (purity >99%) was purchased from JingZhu Biological Technology (Nanjing, China). Uric acid, allopurinol, yeast polysaccharide, and HEPES were purchased from Sigma-Aldrich (United States). Potassium oxonate (PO), rosiglitazone, GW9662, WR-1065, and Pifithrin-β were purchased from MedChemExpress (United States). Antibodies against acetylated-p53, p53 and PPARγ were obtained from Cell Signaling Technology (United States). Antibodies against Lamin A/C, Glyceraldehyde-3-phosphate dehydrogenase (GAPDH), and β-actin were obtained from Santa Cruz Biotechnology (United States). Antibodies against SLC2A9 and ABCG2 were obtained from Novus (United States) and Abcam (United States), respectively. Penicillin/streptomycin and TRIzol reagent were purchased from Invitrogen Life Technologies (United States).

### Animals

Male C57BL/6 mice (6–8 weeks old) were provided by the Academy of Medical Sciences of Zhejiang Province. Mice were given *ad libitum* access to food and water. All experiments were conducted in accordance with the Animal Care and Use Committee guidelines of Zhejiang province. All experimental procedures were approved by the Institutional Animal Care and Use Committee of Zhejiang University.

**FIGURE 1:**
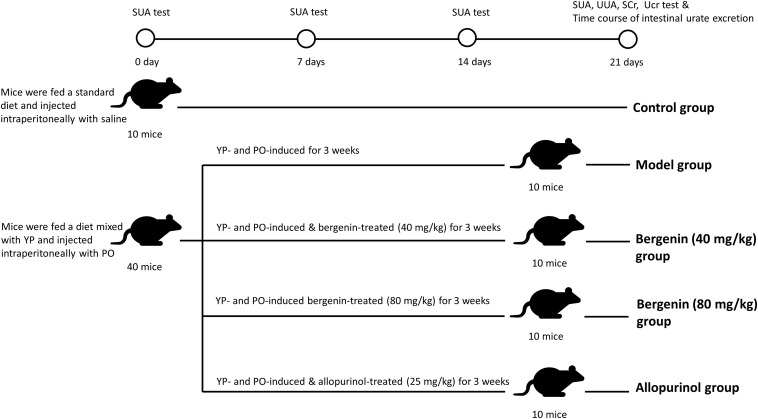
Schematic representation of the procedures followed to develop a hyperuricemia mouse model. Control mice were fed with a standard diet and were intraperitoneally injected with saline daily. To induce hyperuricemia, mice were fed with food supplemented with 25% yeast polysaccharide (YP) and were intraperitoneally injected with potassium oxonate (PO, 250 mg/kg) daily. Mice in the model group were subjected to intragastrical injections of saline daily. In the treatment groups, mice received intragastrical injections of different concentrations of bergenin. Mice in the positive control group were administered allopurinol (25 mg/kg) daily.

Control mice were fed a standard diet. To induce hyperuricemia, mice were given 25% yeast polysaccharide (YP) mixed in daily diet and intraperitoneal injected of PO (250 mg/kg) at 8:00 a.m. every day. Mice of the treatment group were subjected to intragastric administration of either of two concentrations (40 or 80 mg/kg) of bergenin, while the same volume of normal saline (NS) was used as a control. Intragastric administration of 25 mg/kg allopurinol was performed as a positive control (Figure 1).

### Measurement of Uric Acid and Creatinine in Serum and Urine

To measure the serum uric acid levels (SUA), we collected blood samples 2 h after treatment on days 0, 7, 14, and 21. The day before sacrificing, urine samples were collected from mice with metabolic changes within 24 h. Mice were sacrificed 2 h after the last treatment, and blood samples were collected. Uric acid and creatinine levels in serum and urine were determined using the phosphotungstic acid method and a Jaffe reaction kit, respectively (Nanjing Jiancheng Biological Technology Co., Ltd., China). Fractional excretion of urate (FEur) was calculated as per a previously reported method (Perez-Ruiz et al., 2002): FEur = (Uur Scr)/(Sur Ucr) × 100%. Sur, serum urate level; Scr, serum creatinine level; Uur, Urinary urate level; Uur, Urinary creatinine level.

### Transintestinal Urate Transport Analysis

Intestinal urate excretion was determined according to a previously described method (Ichida et al., 2012). Briefly, after overnight fasting, mice were anesthetized with 2% isoflurane inhalation using an isoflurane delivery system 2 h after the last treatment. Subsequently, mice were cannulated with polyethylene tubing at the upper duodenum and middle jejunum, making an intestinal loop at the upper half of the intestine. The intestinal contents were slowly removed by saline and air. Efflux buffer (saline containing 0.3 mM PO) was added into the intestinal loop; the bugger was collected at the indicated time points, and urate concentrations were quantified. Intestinal urate excretion was calculated using the following equation: intestinal urate excretion = (urate concentration in the intestinal loop) × (volume of efflux buffer in the intestinal loop) (length of the whole small intestine/length of the intestinal loop). Urate concentration was determined using the QuantiChrom Uric Acid Assay Kit (Bioassay Systems, United States).

### Histological Examination

After mice were sacrificed, kidney tissues were collected and cleaned, followed by fixation in 4% paraformaldehyde for at least 48 h at room temperature. Specimens were embedded in paraffin wax, and 3-mm thick sections were prepared. After mounting the sections onto polylysine-coated slides, hematoxylin and eosin, as well as Masson’s stainings were performed on consecutive tissue sections. Images were obtained using a microscope.

### Xanthine Oxidase Activity Measurement

Xanthine oxidase (XO) activity in liver tissues was measured using a Xanthine Oxidase Activity Assay Kit (Sigma, United States) according to the manufacturer’s instructions. XO activity was determined by a coupled enzyme assay, which results in a colorimetric (570 nm) fluorometric product, which is proportional to the hydrogen peroxide generated. XO activity was expressed as nanomoles of uric acid per min per mg of total protein (mU/mg).

### Cytokine Measurement

Blood samples were collected 2 h after the last treatment. The concentrations of IL-1β, TNFα, IL-6, IL-10, and IL-1Ra were determined using the respective ELISA kits (BOSTER, China for IL-1Ra and NEOBIOSCIENCE, China for the rest) according to the manufacturer’s instructions.

### Cell Culture

HK-2, Caco-2, and RAW264.7 cells were kindly provided by Stem Cell Bank, Chinese Academy of Sciences (Shanghai, China). HK-2 cells were maintained in Dulbecco’s modified Eagle medium (DMEM)/F12 medium (Gibco, United States) containing 10% fetal bovine serum (FBS; Gibco, Australia). Caco-2 and RAW264.7 cells were cultured in high-glucose DMEM (Gibco, United States) supplemented with 10% FBS (Gibco, Australia). Cells were maintained in a humidified incubator containing 5% CO_2_ at 37°C.

Inhibitors were dissolved in DMSO or double-distilled water (ddH_2_O). Prior to treatments, we cultured cells overnight in serum-free medium to induce growth arrest. Cells were treated with bergenin and indicated inhibitors in a humidified incubator containing 5% CO_2_ at 37°C, with or without stimulation with soluble uric acid for an additional 12 h. The final concentrations and incubation times were as follow: rosiglitazone (30 μM, 2 h), GW9662 (10 μM, 2 h), WR-1065 (1 mM, 2 h), and Pifithrin-β (10 μM, 2 h). Following the addition of HEPES at a final concentration of 25 mM, cells were treated with uric acid or the solvent (10 mM NaOH). The solution was filtered through a 0.22-μm pore size filter (Millipore, Shanghai, China) before use.

### CCK-8

The effect of bergenin on the viability of HK-2 and Caco-2 cells was evaluated using the CCK8 assay. Cells were treated with different concentrations of bergenin, followed by incubation with 10% CCK-8 (Dojindo, Kumamoto, Japan) in 100 μL of high-glucose serum-free DMEM for 4 h at 37°C. Absorbance at 450 nm was measured on a microplate reader (ELX808; BioTek, Winooski, VT, United States).

### Extraction of Subcellular Fractions

For total protein extraction, cells were washed with ice-cold phosphate-buffered saline (PBS) and lysed in radioimmunoprecipitation assay (RIPA) lysis buffer supplemented with proteasome inhibitors (Beyotime, Shanghai, China).

Nuclear and cytoplasmic extracts were prepared using the NE-PER Nuclear Cytoplasmic Extraction Reagent Kit (Pierce, Rockford, IL, United States) according to the manufacturer’s instructions. Briefly, cells were washed in PBS. Ice-cold CER I buffer was added to the cell pellet and vortexed vigorously for 15 s. After a 10-min incubation on ice, ice-cold CER II buffer was added to the cells. Samples were incubated on ice for 1 min, followed by centrifugation for 5 min at 16,000 × *g.* Subsequently, the supernatant containing the cytoplasmic extract was immediately transferred to a pre-chilled tube.

### Western Blot Analysis

Equal amounts of protein were separated by 8–12% sodium dodecyl sulfate-polyacrylamide gel electrophoresis and transferred onto polyvinylidene fluoride membranes (Millipore). Membranes were blocked in 5% non-fat dry milk for 2 h at room temperature, followed by overnight incubation at 4°C with the appropriate primary antibody: GAPDH (1:2000), ABCG2 (1:1000), SLC2A9 (1:1000), PPARγ (1:1000), p53 (1:1000), acetylated-p53, β-actin (1:1000), or Lamin A/C (1:1000). Horseradish peroxidase (HRP)-conjugated goat anti-rabbit or goat anti-mouse IgG (1:5000; Cell Signaling Technology) secondary antibody was applied for 1 h at room temperature. Membranes were covered with enhanced chemiluminescence solution (Millipore) and exposed to film. Signal intensity was measured using the Bio-Rad XRS chemiluminescence detection system (Bio-Rad, Hercules, CA, United States).

### Immunofluorescence

HK-2 and Caco-2 cells were seeded onto 24-well plates. After treatment, cells were fixed in 4% paraformaldehyde for 15 min, washed with PBS, and permeabilized with or without 0.1% Triton X-100 (Beyotime) for 30 min. After blocking in 10% goat serum for 60 min, slides were incubated with a rabbit p53 antibody (1:200) overnight at 4°C. Samples were then incubated with Alexa Fluor 488-conjugated goat anti-rabbit IgG antibody (Invitrogen) for 2 h, and nuclei were stained with 4′,6-diamidino-2-phenylindole (DAPI, Sigma, United States). Samples were observed under a fluorescence microscope (Leica, Solms, Germany).

### Real-Time Quantitative Polymerase Chain Reaction (RT-qPCR)

Total RNA was isolated using TRIzol reagent (Invitrogen) and quantified by measuring the absorbance at 260 nm (NanoDrop 2000; Thermo Fisher Scientific, Waltham, MA, United States). Complementary single-stranded DNA was synthesized from total RNA by reverse transcription (PrimerScript RT Master Mix, TaKaRa, Kyoto, Japan). RT-qPCR reactions were prepared using the SYBR Premix Ex Taq Kit (TaKaRa) in a total volume of 20 μL. All reactions were prepared in duplicates and were run on an ABI StepOnePlus System (Applied Biosystems, Warrington, United Kingdom). The PCR temperature cycling conditions were as follows: 95°C for 30 s followed by 40 cycles at 95°C for 5 s and 60°C for 30 s. Relative gene expression was analyzed using the 2^–ΔΔCt^ method. The primer sequences used are provided in Supplementary Table S1.

### Transfection of Cells With Small Interfering RNA (siRNA)

Cells were seeded onto 6-well plates and cultured overnight in DMEM/F12 or DMEM without FBS and antibiotics. siRNA transfections were carried out using Lipofectamine 2000 (Invitrogen) according to the manufacturer’s instructions. Briefly, 10 μL of siRNA and 5 μL of Lipofectamine 2000 reagent were combined in a total of 300 μL of Opti-MEM I (Gibco, Invitrogen). Thereafter, 700 μL of Opti-MEM I was added to the mixture, and the mixture was added to each well. After incubation for 6 h, fresh DMEM or DMEM/F12 containing 10% FBS was added to each well. Cells were incubated for an additional 48–72 h. *PPAR-*γ siRNAs and the negative control siRNAs were purchased from GenePharma (Shanghai, China).

### Luciferase Reporter Assay

Cells were transfected with luciferase reporter constructs *PPAR*γ-Luc (Promega, United States) or *p53*-Luc (Promega, United States) as previously described (Ye et al., 2019). Cells were then treated with uric acid (UA, 8 mg/dL) and/or bergenin for 12 h. Subsequently, luciferase activity was measured using a luciferase assay system (Promega, United States).

### Statistical Analysis

Statistical analysis was performed using the SPSS statistical software for Windows, version 19.0 (IBM, Armonk, NY, United States). All experiments were performed at least in triplicate, and the data were expressed as mean ± standard error of the mean (SEM). Statistical significance was determined using one-way analysis of variance (ANOVA) followed by Fisher’s Least Significant Difference (LSD) test when comparing more than two groups. *P* values ≤ 0.05 were considered statistically significant. The correlation between variables was evaluated by the Pearson correlation test. Two-sided *P* values < 0.05 were considered statistically significant.

## Results

### Bergenin Ameliorates Hyperuricemia in Mice Treated With Potassium Oxonate (PO) and Yeast Polysaccharide (YP)

**FIGURE 2:**
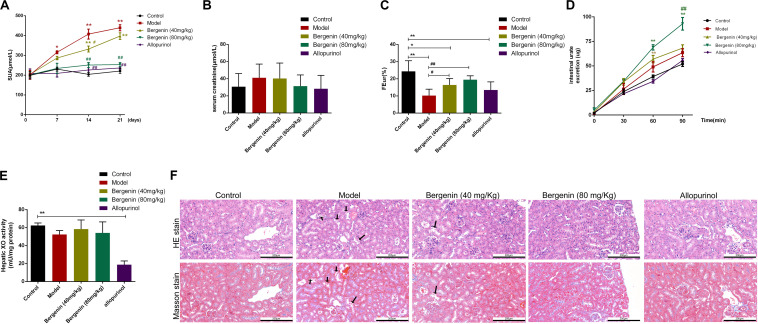
Effects of bergenin on hyperuricemia in mice treated with PO and YP. **(A–E)** Serum uric acid (SUA) levels **(A)**, serum creatinine levels (Scr; *n* = 10) **(B)**, fractional excretion of urate (FEur) (*n* = 6) **(C)**, time course of intestinal urate excretion (*n* = 3) **(D)**, and hepatic xanthine oxidase (XO) activity **(E)** in the different groups. Data are presented as mean ± SEM. *P < 0.05 and **P < 0.01, compared to the control group; ^#^*P* < 0.05 and ^##^*P* < 0.01, compared to the model group. **(F)** Histopathological analysis of kidney tissues after H&E and Masson’s staining (magnification, ×200). Scale bar = 200 μm Degeneration and necrosis in renal tubular epithelial cells are indicated by black arrows.

The procedure followed to develop a hyperuricemia mouse model is illustrated in Figure 1. Compared with control mice, SUA levels were significantly higher in hyperuricemia mice after day 7, and remained elevated between day 14 (407.41 ± 79.09 μmol/L vs. 194.07 ± 25.85 μmol/L; *P* < 0.01) and day 21 (439.39 ± 48.11 μmol/L vs. 220.60 ± 35.28 μmol/L; *P* < 0.01). SUA elevation was suppressed by bergenin (80 mg/kg) and allopurinol administration. While bergenin at 80 mg/kg profoundly decreased SUA levels (253.18 ± 31.74 μmol/L) compared to hyperuricemia mice, no significant difference was observed at day 21 after administration of 40 mg/kg bergenin (397.39 ± 52.69 μmol/L; Figure 2A). This finding suggests that the effects of bergenin are dose-dependent to some degree. It is worth noting that SUA level of the Bergenin (80 mg/kg) group stabilized at baseline levels from day 1 to day 21. As expected, SUA levels were significantly lower in allopurinol-treated mice (positive control) compared with control mice. No significant differences in SUA levels were observed between the mice in the bergenin (80 mg/kg) group and the allopurinol group.

Scr, FEur, intestinal urate excretion rate, and hepatic XO activity were assessed at day 21. Scr levels were higher in hyperuricemia mice compared with control mice; however, the difference did not reach statistical significance (*P* > 0.05) (Figure 2B). In hyperuricemic mice, FEur was decreased; bergenin (40 mg/kg, 80 mg/kg) treatment rescued FEur. Furthermore, urate excretion from the intestine was significantly increased in mice treated with bergenin (80 mg/kg) compared with hyperuricemic mice (*P* < 0.01; Figure 2D). While allopurinol reduced hepatic XO activity (*P* < 0.01), it did not affect uric acid excretion in the kidneys and intestine (*P* > 0.05) compared with hyperuricemia mice (Figures 2C–E). Additionally, bergenin had no effect on XO activity in liver and jejunum (Figure 2E, Supplementary Figure S1).

Histological analysis revealed that the ultrastructure of the kidneys was intact in mice treated with PO and YP. Compared with control mice, degeneration and necrosis were observed in tubular epithelial cells of hyperuricemic mice (Figure 2F). Bergenin (80 mg/kg) and allopurinol treatment ameliorated PO- and YP-induced pathological lesions. No histological changes were observed in the intestine of hyperuricemic or bergenin-treated mice (Supplementary Figure S2).

### Bergenin Regulates the Expression of Urate Transporters in the Kidney

**FIGURE 3:**
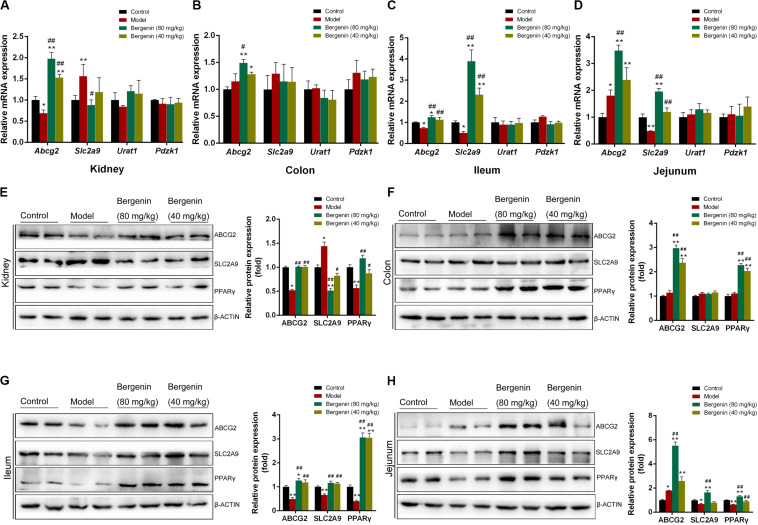
Effects of bergenin on urate transporters in the kidney and intestine. **(A)** Relative mRNA levels of ABCG2, SLC2A9, URAT1, and PDZK1 in the kidneys determined by RT-PCR. **(B–D)** Relative mRNA levels of ABCG2, SLC2A9, PDZK1, and SLC17A3 in the colon **(B)**, ileum **(C)**, and jejunum **(D)** determined by RT-PCR. **(E–H)** Representative western blot analysis showing ABCG2, SLC2A9, and PPARγ protein levels in the kidneys **(E)**, colon **(F)**, ileum **(G)**, and jejunum **(H)**, and quantitative analysis of the band intensities. Protein levels were normalized to GAPDH. Data are presented as mean ± SEM. *P < 0.05 and **P < 0.01, compared to the control group; ^#^*P* < 0.05 and ^##^*P* < 0.01 compared to the model group; *n* = 4–6.

To determine the effects of bergenin on uric acid excretion in the kidneys, we analyzed the expression of urate transporters involved in urate export and reuptake in the kidney. We found no significant differences in the mRNA levels of the genes encoding urate transporter 1 (*Urat1*) and PDZ domain-containing 1 (*Pdzk1*) among the different groups (*P* > 0.05; Figure 3A). *Abcg2* expression was lower in hyperuricemic mice compared with control mice; nevertheless, its expression in hyperuricemic mice was rescued by bergenin (40 mg/kg, 80 mg/kg) treatment, both at the mRNA and protein levels (*P* < 0.01) (Figures 3A,E). Bergenin at 80 mg/kg and 40 mg/kg resulted in 2-fold and 1.5-fold increases in *Abcg2* mRNA levels, respectively.

In contrast, *Slc2a9* expression was higher in hyperuricemic mice compared with control mice, and bergenin (80 mg/kg) suppressed *Slc2a9* expression, which was evident both at the mRNA and protein levels (*P* < 0.01). Although bergenin treatment (40 mg/kg) decreased SLC2A9 protein levels (*P* < 0.05, compared to the model group), no difference was observed at the mRNA level (Figures 3A,E). Hyperuricemic mice exhibited a reduction in PPARγ expression (*P* < 0.01, compared to control mice), PPARγ expression was restored by bergenin (40 mg/kg, 80 mg/kg; Figure 3E).

### Bergenin Regulates the Expression of Urate Transporters in the Intestine

RT-qPCR analyses revealed no significant differences in the expression of *Pdzk1* and solute carrier family 17 member 3 (*Slc17a3*) in the intestine among the groups (*P* > 0.05; Figures 3B–D). In the colon, bergenin (40 mg/kg, 80 mg/kg) significantly increased *Abcg2* mRNA and protein levels (Figures 3B,F). Hyperuricemic mice had lower *Abcg2* mRNA and protein levels compared with the control group, which were restored by bergenin (40 mg/kg, 80 mg/kg) treatment (Figures 3C,G). In the jejunum, the expression of *Abcg2* was increased in hyperuricemic mice, and its expression was elevated by bergenin in a dose-dependent manner, both at the mRNA and protein levels (Figures 3D,H). These RT-qPCR and western blot findings were also confirmed by immunofluorescence (Supplementary Figure S3).

Compared to control mice, hyperuricemic mice exhibited decreased expression of *Slc2a9* in the ileum and jejunum, both at the mRNA and protein levels (*P* < 0.01). Administration of bergenin significantly restored *Slc2a9* expression in the ileum and jejunum (Figures 3C,D,G,H). The mRNA levels of *Slc2a9* were increased by approximately 2-fold and 4-fold after administration of bergenin at 40 and 80 mg/kg, respectively, suggesting a dose-dependent effect. On the other hand, RT-qPCR and western blot analyses showed no changes in *Slc2a9* expression in the colon (Figures 3B,F). SLC2A9 expression in intestine was further confirmed by immunofluorescence (Supplementary Figure S3).

PPARγ was expressed at lower levels in the ileum and jejunum of hyperuricemic mice (*P* < 0.01, compared with control mice), but not in the colon. Bergenin treatment enhanced PPARγ expression in a dose-dependent manner (Figures 3F–H).

### Bergenin Reduces Pro-inflammatory Cytokine Serum Levels

**FIGURE 4:**
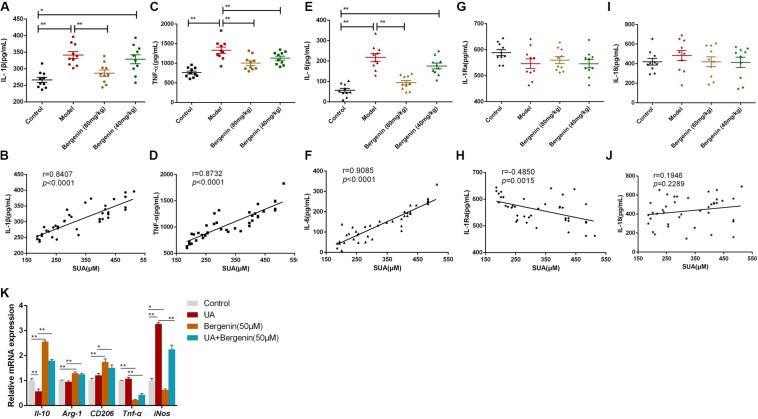
Effects of bergenin on the serum levels of inflammatory cytokines. Serum levels of IL-1β **(A)**, TNF-α **(C)**, IL-6 **(E)**, IL-1Ra **(G)**, and IL-18 **(I)** were measured by ELISA. Data are presented as mean ± SEM. *P < 0.05 and **P < 0.01; *n* = 10. Correlation analysis between SUA levels and serum levels of the pro-inflammatory cytokines IL-1β **(B)**, TNF-α **(D)**, IL-6 **(F)**, IL-1Ra **(H)**, and IL-18 **(J)**. **(K)** mRNA levels of IL-10, Arg-1, TNF-α, and iNOS in RAW-264.7 cells. Data are presented as mean ± SEM. *P < 0.05 and **P < 0.01.

Several studies suggested that hyperuricemia and soluble uric acid levels are associated with systemic inflammation (Joosten et al., 2020). Therefore, we investigated the effects of bergenin on the serum levels of several inflammatory cytokines. IL-1β, TNF-α, and IL-6 serum levels were significantly increased in hyperuricemic mice; bergenin treatment reduced the serum levels of these cytokines. Notably, bergenin at 80 mg/kg reduced the serum levels of IL-1β, TNF-α, and IL-6 by 16%, 25%, and 57%, respectively (Figures 4A,C,E). In contrast, no significant difference was observed in the serum levels of IL-1Ra or IL-18 (Figures 4G,I). Additionally, SUA levels were positively correlated with IL-1β, TNF-α and IL-6 serum levels and were negatively correlated with those of IL-1Ra (Figures 4B,D,F,H). No correlation between SUA and IL-18 (Figure 4J).

To gain further insight into the effects of bergenin on inflammatory responses, we treated RAW267.4 cells with UA (8 mg/dL) or bergenin (50 μM). Exposure to UA polarized RAW267.4 cells toward an inflammatory (M1) phenotype with upregulation of inducible nitric oxide synthase (*iNos*) and downregulation of *Il-10*. In contrast, bergenin treatment polarized RAW267.4 cells toward an anti-inflammatory (M2) phenotype, with high expression of Arginase-1 (*Arg-1*), *Il-10*, and *CD206* and low expression of *Tnf-*α and *iNos.* RAW267.4 cells pretreated with bergenin prior to UA exposure acquired an M2 phenotype, with increased *Arg-1, Il-10*, and *CD206* expression and low *Tnf-*α and *iNos* expression (Figure 4K).

### Bergenin *in vitro* Treatment Regulates the Expression of ABCG2 and SLC2A9 in HK-2 Human Renal Proximal Tubular Epithelial Cells

**FIGURE 5:**
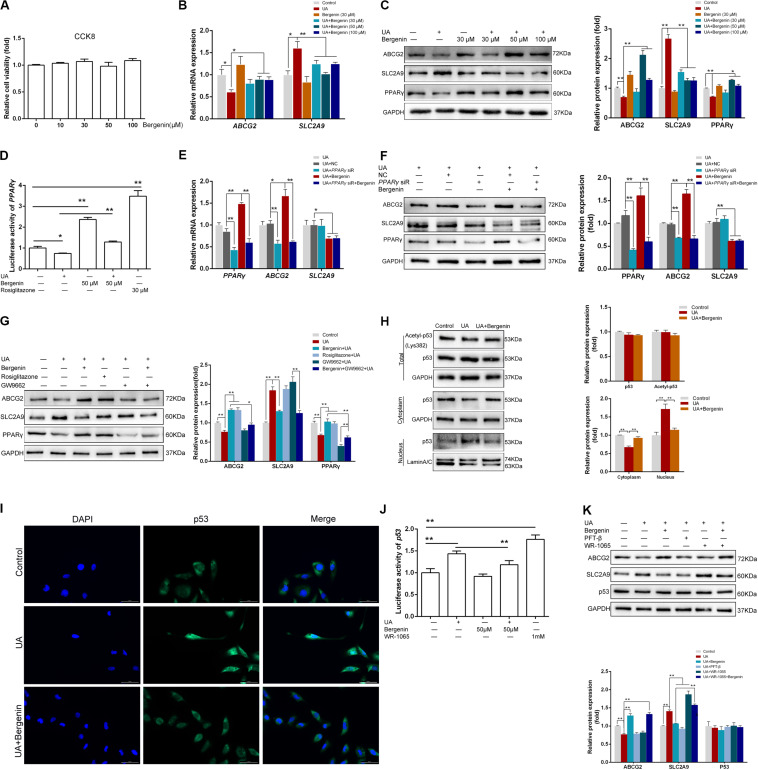
Bergenin regulates the expression of ABCG2 and SLC2A9 in HK-2 cell lines. **(A)** HK-2 cell lines were treated with bergenin (10, 30, 50, or 100 μM) for 12 h, and cell viability was determined by the CCK8 assay. **(B)** HK-2 cell lines were pretreated with bergenin (0, 30, 50, or 100 μM) for 2 h, followed by treatment with 8 mg/dL uric acid (UA) for 10 h. Relative mRNA levels of ABCG2 and SLC2A9 were determined by RT-qPCR. **(C)** Representative western blot showing ABCG2, SLC2A9, and PPARγ protein levels. Protein levels were normalized to GAPDH. **(D)** Transcriptional activity of PPARγ, as determined by luciferase assay. **(E)** Cells were transfected with PPARγ siRNA or scrambled siRNA for 48 h. Cells were then pretreated with or without 50 μM bergenin for 2 h, followed by exposure to 8 mg/dL UA for another 10 h. Relative mRNA levels of ABCG2, SLC2A9, and PPARγ were determined by RT-qPCR. **(F)** Representative western blot showing ABCG2, SLC2A9, and PPARγ protein levels. Protein levels were normalized to GAPDH. **(G)** Cells were pretreated with bergenin (50 μM) or rosiglitazone (PPARγ agonist) for 2 h, followed treatment with 8 mg/dL UA for another 10 h, with or without GW9662 (PPARγ inhibitor). ABCG2, SLC2A9, and PPARγ protein levels were determined by western blotting. Protein levels were normalized to GAPDH. **(H)** Total p53 and acetylated p53 levels were measured by western blot analysis. Protein levels were normalized to GAPDH. Cytoplasmic and nuclear extracts were prepared for western blot analyses. Cytoplasmic protein levels were normalized to GAPDH, whereas nuclear protein levels were normalized to Lamin A/C. **(I)** Representative immunofluorescence images (magnification, ×400) showing p53 expression (green). Nuclei were stained with DAPI (blue). Scale bar = 50 μm. **(J)** Transcriptional activity of p53, as determined by luciferase assay. **(K)** Cells were pretreated with bergenin (50 μM) or the p53 inhibitor Pifithrin-β (PFT-β) for 2 h, followed by treatment with 8 mg/dL UA for another 10 h, with or without WR-1065 (p53 agonist). ABCG2, SLC2A9, and p53 protein levels were determined by western blot analysis. Protein levels were normalized to GAPDH. Data are presented as mean ± SEM. *P < 0.05 and **P < 0.01, *n* = 3.

We found that bergenin at 10–100 μM did not affect the viability of HK-2 cells (Figure 5A). Hence, we pretreated HK-2 cells with 10, 30, 50, or 100 μM bergenin for 2 h, followed by treatment with 8 mg/dL UA for 10 h. UA decreased *ABCG2* expression at the mRNA and protein level (*P* < 0.05, compared to the control group). UA-mediated *ABCG2* downregulation was rescued by bergenin treatment (50 and 100 μM). In contrast, *SLC2A9* mRNA and protein levels were increased after UA treatment (*P* < 0.05, compared to the control group). Pretreatment with bergenin (50 and 100 μM) suppressed UA-induced *SLC2A9* upregulation (Figures 5B,C); however, lower concentrations of bergenin did not affect *SLC2A9* expression. No significant increase was observed in *ABCG2* and *SLC2A9* expression in cells treated with 30 μM bergenin alone (Figures 5B,C).

### Bergenin Promotes ABCG2 Expression by Activating PPARγ in HK-2 Cells

Luciferase reporter assays revealed that UA significantly reduced the expression and transcriptional activity of *PPAR*γ, both of which were restored by bergenin (Figures 5C,D). siRNA-mediated *PPAR*γ silencing decreased the mRNA and protein levels of *ABCG2*. Moreover, bergenin treatment failed to enhance *ABCG2* expression after *PPAR*γ knockdown. *PPAR*γ silencing did not affect the expression of *SLC2A9*, regardless of the presence of bergenin (Figures 5E,F).

Similarly to bergenin, pretreatment of HK-2 cells with the PPARγ agonist rosiglitazone before stimulation with UA increased the expression of PPARγ and ABCG2. In contrast, pretreatment with the PPARγ antagonist GW9662 enhanced the UA-mediated downregulation of PPARγ and ABCG2 (Figure 5G). Additionally, bergenin failed to restore ABCG2 expression in cells treated with GW9662 and UA (Figure 5G). These results suggest that bergenin regulates the expression of ABCG2 by activating PPARγ.

### Bergenin Reduces SLC2A9 Expression by Diminishing Nuclear Translocation of p53 in HK-2 Cells

As the previous research indicated *SLC2A9* was a direct target gene of *p53* (Itahana et al., 2015), we next investigated the effects of bergenin and UA on *p53* signaling. Although bergenin and UA had no effects in the total p53 and acetylated p53 levels (Figure 5H), UA significantly decreased p53 levels in the cytoplasm and increased its levels in the nucleus. These results suggest that UA promotes p53 protein translocation from the cytoplasm to the nucleus. Interestingly, bergenin suppressed nuclear translocation of p53 (Figure 5H). Immunofluorescence and luciferase reporter analyses confirmed these results (Figures 5I,J).

Pifithrin-β (PFT-β), a potent *p53* inhibitor, significantly suppressed the UA-induced SLC2A9 upregulation. On the other hand, the *p53* agonist WR-1065 reinforced SLC2A9 upregulation. Bergenin treatment failed to suppress SLC2A9 expression in HK-2 cells treated with WR-1065 and UA (Figure 5K).

### Bergenin *in vitro* Treatment Promotes the Expression of ABCG2 and SLC2A9 in Intestinal Epithelial Cells

**FIGURE 6:**
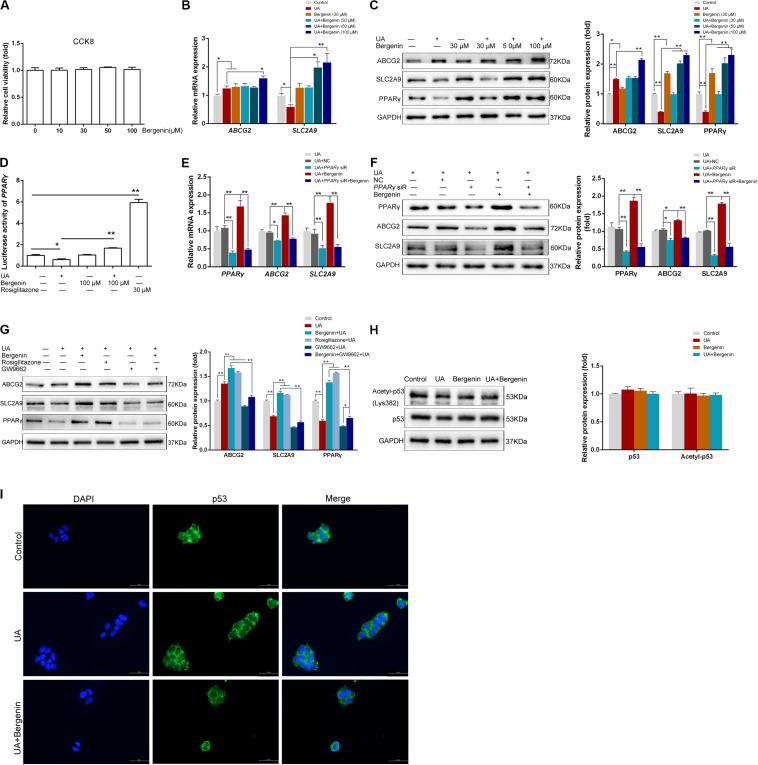
Bergenin regulates the expression of ABCG2 and SLC2A9 in Caco-2 cell lines. **(A)** Cells were treated with bergenin (10, 30, 50, and 100 μM) for 12 h, and cell viability was determined by the CCK8 assay. **(B)** Caco-2 cells were pretreated with 0, 30, 50, or 100 μM bergenin for 2 h, followed by treatment with 8 mg/dL UA for 10 h. Relative mRNA levels of ABCG2, and SLC2A9, as determined by RT-qPCR. **(C)** Representative western blots showing ABCG2, SLC2A9, and PPARγ protein levels. Protein levels were normalized to GAPDH. **(D)** Transcriptional activity of PPARγ, as determined by luciferase assays. **(E)** Cells were transfected with PPARγ siRNA or scrambled siRNA for 48 h. Cells were then pretreated with or without 100 μM bergenin for 2 h, followed by incubation with 8 mg/dL UA for another 10 h. Relative mRNA levels of ABCG2, SLC2A9, and PPARγ, as determined by RT-qPCR. **(F)** Representative western blots showing ABCG2, SLC2A9, and PPARγ protein levels. Protein levels were normalized to GAPDH. **(G)** Cells were pretreated with bergenin (100 μM) or rosiglitazone (PPARγ agonist) for 2 h followed by incubation with 8 mg/dL UA for another 10 h, with or without GW9662 (PPARγ inhibitor). ABCG2, SLC2A9, and PPARγ protein levels were determined by western blotting. Protein levels were normalized to GAPDH. **()H** Total p53 and acetylated p53 levels were determined by western blot analysis. Protein levels were normalized to GAPDH. **(I)** Representative immunofluorescence images (magnification, ×400) showing p53 expression (green). Nuclei were stained with DAPI (blue). Scale bar = 50 μm.

We next used Caco-2 cells as an *in vitro* model of human intestinal epithelial cells to examine the effects of bergenin on intestinal urate transporters. We found that treatment with bergenin at 10–100 μM for 12 h had no effects on the viability of Caco-2 cells (Figure 6A). We then pretreated Caco-2 cells with bergenin for 2 h, followed by treatment with UA at 8 mg/dL for 10 h. UA increased *ABCG2* mRNA and protein levels and downregulated *SLC2A9* levels. Treatment with bergenin alone increased the protein levels of ABCG2 and SLC2A9. Compared to cells treated with UA alone, *ABCG2* and *SLC2A9* were significantly upregulated when cells were treated with UA and 100 μM bergenin (*P* < 0.01) (Figures 6B,C).

### Bergenin Promotes ABCG2 and SLC2A9 Expression by Activating PPARγ in Caco-2 Cells

Similar to HK-2 cells, UA significantly inhibited PPARγ activity in Caco-2 cells, which was restored by bergenin (Figures 6C,D). Interestingly, siRNA-mediated *PPAR*γ silencing reduced the expression of both *ABCG2* and *SLC2A9*. In addition, bergenin treatment failed to enhance *ABCG2* and *SLC2A9* expression after *PPAR*γ silencing (Figures 6E,F). PPARγ activation by rosiglitazone significantly enhanced the UA-mediated ABCG2 and SLC2A9 upregulation (*P* < 0.01). In contrast, pretreatment with GW9662 enhanced the UA-mediated downregulation of PPARγ, ABCG2, and SLC2A9. Furthermore, bergenin failed to restore ABCG2 and SLC2A9 expression in cells treated with GW9662 and UA (Figure 6G).

### Bergenin Does Not Affect the p53 Signaling Pathway in Caco-2 Cells

Bergenin treatment did not alter the total p53 and acetylated p53 levels in Caco-2 cells (Figure 6H). Additionally, immunofluorescence analyses indicated that bergenin did not promote nuclear translocation of p53 (Figure 6I).

## Discussion

As serum urate levels are associated with the development of gout, interventions that reduce serum urate concentrations have been investigated for their potential to prevent gout (Dalbeth et al., 2019). In this study, we assessed the effect of bergenin on hyperuricemia using a mouse model. We showed for the first time that bergenin has uricosuric properties, reducing serum urate levels in hyperuricemic mice. We also provided evidence that its urate-lowering effects are mediated by increasing *Abcg2* expression in the kidneys and intestine, as well as *Slc2a9* downregulation in the kidneys. Furthermore, we demonstrated that bergenin downregulates IL-6, IL-1β, and TNF-α serum levels in hyperuricemic mice and polarizes RAW264.7 cells toward an M2 phenotype.

ABCG2 is a high-capacity urate exporter expressed in the intestine and kidneys, and ABCG2 dysfunction strongly increases serum urate levels and the risk of gout development (Ichida et al., 2012; Dalbeth et al., 2019). In this study, we found that although *Abcg2* expression was increased in the jejunum, its levels were decreased in the kidneys and ileum of hyperuricemic mice. After treatment with bergenin, *Abcg2* was significantly upregulated in the kidneys, jejunum, ileum, and colon, enhancing renal and intestine urate excretion. These findings may partly explain the anti-hyperuricemic effects of bergenin. *PPAR*γ is a ligand-regulated transcription factor involved in various pathophysiological processes, including metabolism, inflammatory responses, and tumorigenesis (Berger and Moller, 2002; Mandard and Patsouris, 2013). Herein, we showed that *PPAR*γ expression was decreased under high-UA conditions both *in vitro* and *in vivo*. Importantly, bergenin restored *ABCG2* expression by activating *PPAR*γ. These findings suggest that *PPAR*γ enhances *ABCG2* expression, which is consistent with previous studies (Szatmari et al., 2006; Wang et al., 2016). It is noteworthy that in Caco-2 cells, although PPARγ was decreased after treatment with UA, *ABCG2* expression was upregulated. This could be explained by the fact that UA is not a specific *PPAR*γ antagonist. In a previous study, we showed that soluble UA induced *ABCG2* expression in Caco-2 cells via the TLR4-NLRP3 inflammasome and PI3K/Akt signaling pathways (Chen et al., 2018). Additionally, the *ABCG2* is the only associated locus with the early onset and/or a family history (Stiburkova et al., 2019). And it has been reported (Roberts et al., 2017) *ABCG2* rs2231142 predicts a poor response to first-line ULT, allopurinol. We will verify the impact of bergenin in the case of *ABCG2* variants with reduced function in the future.

*SLC2A9* is widely expressed, including in the liver, kidneys, intestine, brain, placenta, lungs, and peripheral leucocytes (Phay et al., 2000; DeBosch et al., 2014). In kidneys, SLC2A9 is responsible for reabsorption of urate at the basolateral membrane in the proximal renal tubule (Dinour et al., 2010). Herein, we demonstrated that bergenin decreased *SLC2A9* expression by inhibiting the nuclear translocation of p53, which indicated that bergenin might not only have effect on urate excretion, but also urate reabsorption in the kidneys. Consistently, Itahana et al. (2015) showed that *SLC2A9* was a direct target gene of the tumor suppressor *p53*. Interestingly, we found no association between *SLC2A9* and *p53* levels in Caco-2 cells. Caco-2 is a well-characterized human colon adenocarcinoma cell line widely used to investigate mechanisms of drug absorption or characterize intestinal transporters. Caco-2 cells harbor *p53* mutations (Lee et al., 2008; Sun et al., 2008), which is a limitation of using this cell line model to assess the relevance of *p53* pathway in the anti-hyperuricemic effects of bergenin. In addition to the liver and kidneys, *SLC2A9* is also expressed on the apical and basolateral gut enterocyte membranes in mice (Lu et al., 2019). Gut enterocyte-specific *Slc2a9*-knockout mice exhibited increased serum urate levels with impaired enterocyte urate transport kinetics. Moreover, these mice developed early onset metabolic syndromes, including hypertension, dyslipidemia, and hyperinsulinemia, suggesting a role of *Slc2a9* in regulating enterocyte urate clearance (DeBosch et al., 2014). The upregulation of *Slc2a9* in the jejunum and ileum reported herein could have contributed to increased urate excretion from the intestine observed after bergenin treatment.

We also detected the xanthine oxidase activity in ileum and jejunum besides livers. The xanthine oxidase activity was almost undetectable in the ileum. No significant difference were found in the jejunum among the groups (Supplementary Figure S1B). However, Yoon et al. (2016) have shown *in vivo* XO inhibitory and antihyperuricemic effects of Corylopsis coreana Uyeki, containing and identified bergenin as one of constituents. As a herbal medicine, Corylopsis coreana Uyeki might also include some other constituents responsible for the XO inhibitory activity. Synergizing action of a combination of several active components may be another possibility.

The crosstalk between *PPAR*γ and *SIRT1* plays an important role in the regulation of metabolism and inflammation (Han et al., 2010; Chou et al., 2017). Furthermore, p53 acetylation at Lys382, the primary target site of *SIRT1*, increases the ability of p53 to activate transcription (Feng et al., 2015; Nakamura et al., 2017). Therefore, we used siRNAs targeting *SIRT1* to explore the interaction. *SIRT1* silencing did not affect the expression of PPARγ, ABCG2, or SLC2A9 in HK-2 and Caco-2 cells, regardless of exposure to bergenin (Supplementary Figure S4).

Increasing evidence suggests that not only crystalline urate, but soluble urate could promote metabolic inflammation, activate innate immunity, and trigger epigenetic alterations that amplify pro-inflammatory responses (Crişan et al., 2017; Joosten et al., 2020). Studies in patients with asymptomatic hyperuricemia have shown that increased serum urate concentrations are associated with inflammatory responses, including increased IL-6, IL-1β, TNF-α, and IL-18 levels and decreased IL-1Ra and IL-10 levels (Crisan et al., 2016; Crişan et al., 2017). Kim et al. (2015) reported that hyperuricemia induced NLRP3 activation in macrophages, accelerated macrophage recruitment, and promoted M1 phenotype polarization, contributing to diabetic nephropathy progression. These findings suggest that in addition to urate-lowering therapies, immunomodulatory therapies may also be helpful in hyperuricemia treatment. Bergenin is a plant-derived compound with well-documented anti-inflammatory properties (Veerapur et al., 2012). Notably, bergenin inhibited collagen-induced arthritis and ameliorated experimental colitis in mice by reducing the serum levels of IL-2, IL-6, and TNF-α (Jain et al., 2014; Wang et al., 2017; Lopes de Oliveira et al., 2019). In this study, we found that the serum levels of IL-1β, TNF-α, and IL-6 were increased in hyperuricemic mice and positively correlated with SUA. Bergenin treatment reduced the serum levels of IL-1β, TNF-α, and IL-6 in hyperuricemic mice.

M1 macrophages are characterized by TNF-α and iNOS expression and mediate tissue damage and initiate inflammatory responses by secreting high levels of various pro-inflammatory cytokines, including IL-1α, TNF-α, and IL-1β (Shapouri-Moghaddam et al., 2018). To protect against excessive tissue damage and inflammation, macrophages can acquire an M2 phenotype, expressing several anti-inflammatory molecules, such as IL-10 and IL-1Ra (Shapouri-Moghaddam et al., 2018). In this study, we showed that bergenin treatment *in vitro* induced *Il-10*, *CD206* and *Arg-1* expression in macrophages while decreasing *Tnf-*α and *iNos* expression at the same time, suggesting a shift from the M1 to M2 phenotype. M2 polarization in macrophages after bergenin treatment can, therefore, be one of the mechanisms responsible for the anti-inflammatory effects of bergenin reported in previous studies. Nevertheless, the in-depth mechanisms involved in the inflammatory effects of bergenin in hyperuricemia need further investigation.

## Conclusion

**FIGURE 7:**
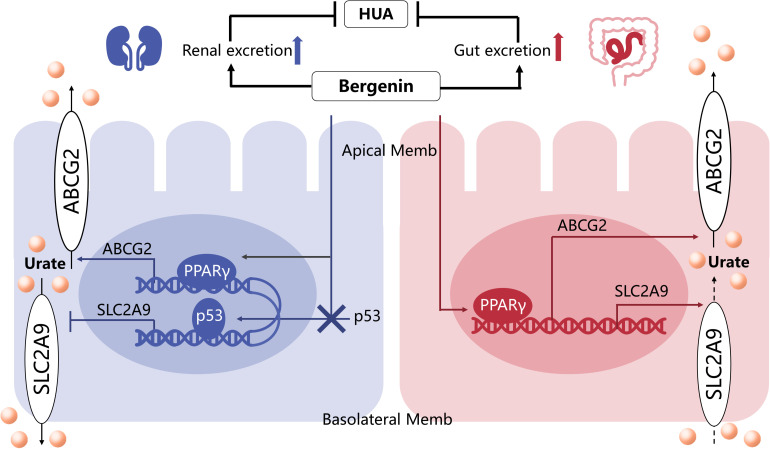
The mechanisms underlying the anti-hyperuricemic effects of bergenin. Bergenin promotes ABCG2 expression in the kidneys and intestine, as well as SLC2A9 expression in the intestine, by activating PPARγ. Moreover, bergenin suppresses SLC2A9 expression in the kidneys by preventing nuclear translocation of p53. Hence the uricosuric and anti-hyperuricemic properties of bergenin are facilitated by uric acid excretion in the kidneys and the gut.

In summary (Figure 7), these findings indicated that bergenin not only can promote renal and gut uric acid excretion via regulating the expression of *ABCG2* and *SLC2A9* but also attenuate inflammation and induce a macrophage polarization shift from the M1 phenotype to M2. Thus, bergenin is a promising candidate as a novel therapeutic strategy for hyperuricemia, either for supplement of the existing ULT or potential intervention in metabolic inflammation.

## Data Availability Statement

The raw data supporting the conclusions of this article will be made available by the authors, without undue reservation, to any qualified researcher.

## Ethics Statement

The animal study was reviewed and approved by Institutional Animal Care and Use Committee of Zhejiang University.

## Author Contributions

HW and XL contributed to design and funding sources to this study. MC and CY drafted the manuscript. MC, JZ, PZ, and YJ did all the *in vitro* parts of the study. All authors have contributed significantly and read and approved the final manuscript.

## Conflict of Interest

The authors declare that the research was conducted in the absence of any commercial or financial relationships that could be construed as a potential conflict of interest.
