# Supplementary figures and images for: Bergenin as a Novel Urate-Lowering Therapeutic Strategy for Hyperuricemia
Source: Front Cell Dev Biol. 2020 Jul 29;8:703. doi: 10.3389/fcell.2020.00703 (PMC7403512; doi:10.3389/fcell.2020.00703)

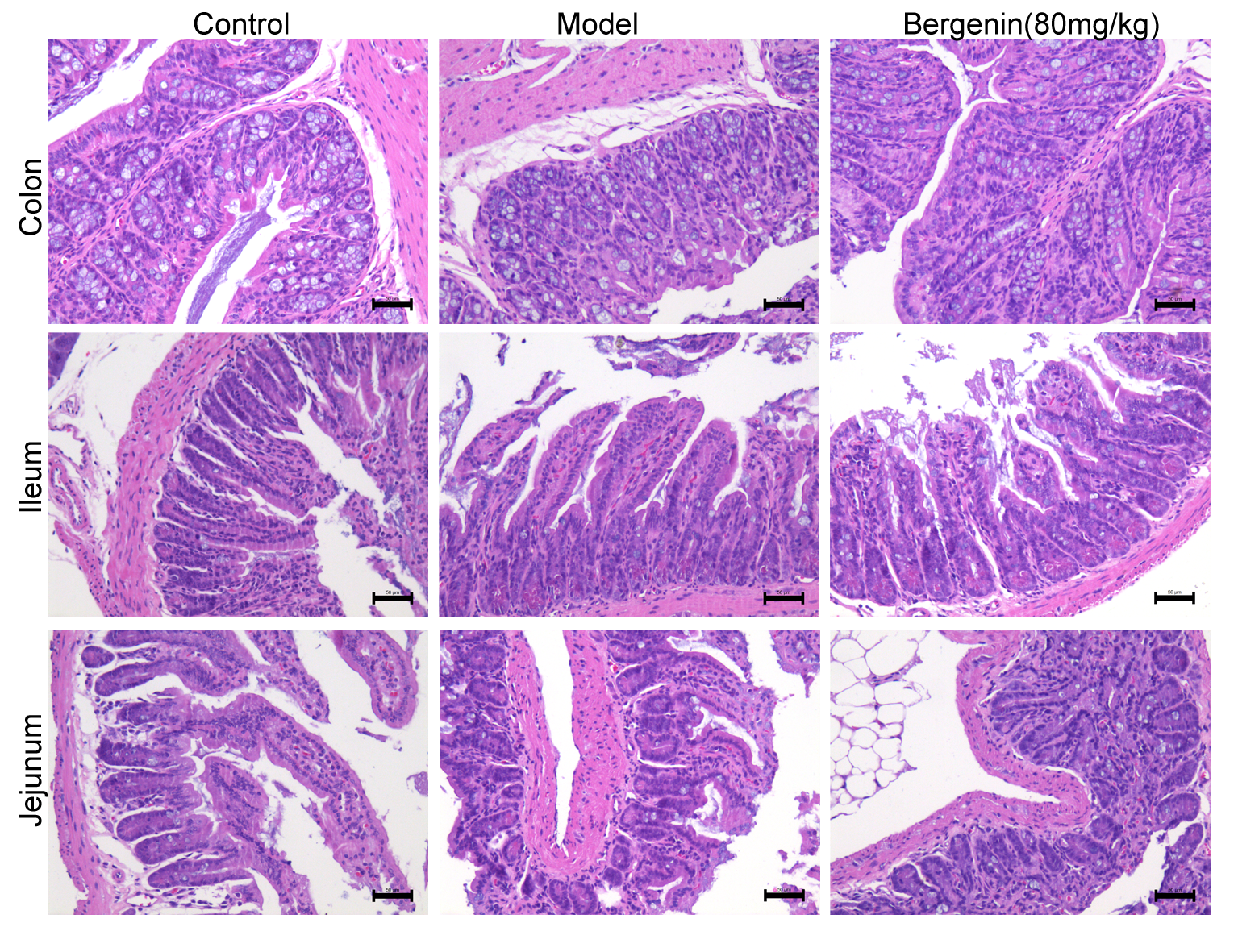

Supplement: Supplementary file 2 [file Image_2.TIF]

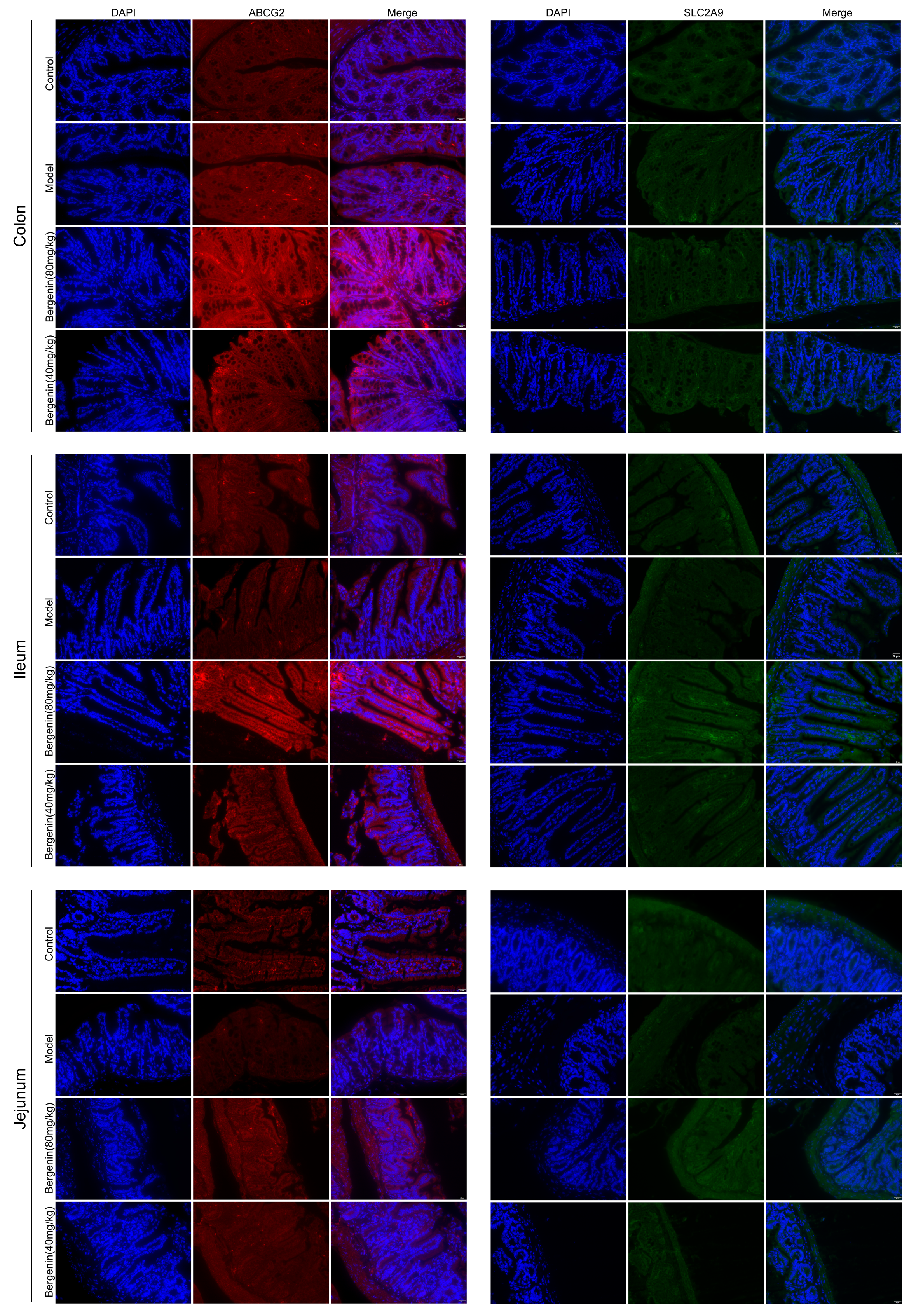

Supplement: Supplementary file 3 [file Image_3.TIF]

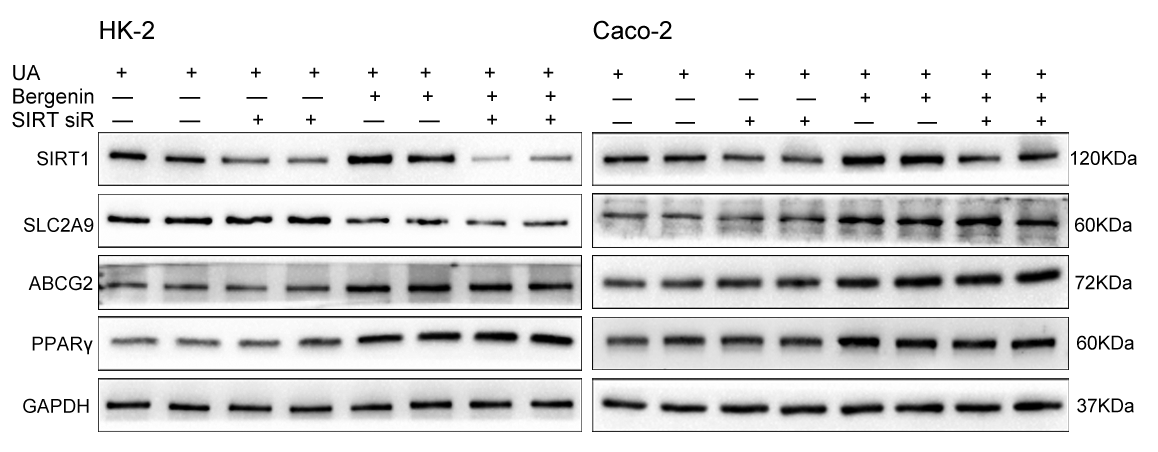

Supplement: Supplementary file 4 [file Image_4.TIF]
