# Supplementary material for: Bergenin as a Novel Urate-Lowering Therapeutic Strategy for Hyperuricemia
Source: Front Cell Dev Biol. 2020 Jul 29;8:703. doi: 10.3389/fcell.2020.00703 (PMC7403512; doi:10.3389/fcell.2020.00703)
Supplement: Supplementary file 5 [file Table_1.DOCX]

Primers used in real-time quantitative polymerase chain reaction

| Human | 5’-3’ |
| --- | --- |
| *GAPDH* | Fwd: AACTCCCACTCTTCCACCTTCG  Rev: TCCACCACCCTGTTGCTGTAG |
| *SLC2A9* | Fwd: CAATAGACCCAGACACTCTGACT  Rev: TCTTCACAATTAACGTCCCCAC |
| *ABCG2* | Fwd: AATACATCAGCGGATACTA  Rev: AATAAGCCACCATCATAAG |
| *PPARγ* | Fwd: GTACTGTCGGTTTCAGAAGTGCC  Rev: ATCTCCGCCAACAGCTTCTCCT |
| Mouse |  |
| Gapdh | Fwd: GACATTTGAGAAGGGCCACAT  Rev: CAAAGAGGTCCAAAACAATCG |
| *Slc2a9* | Fwd: ATTCCAACCACTTGCTCTCCC  Rev: GGACCATTTCCGCTGGCTTTC |
| *Abcg2* | Fwd: AAATGCTGTTCAGGTTATGTGGT  Rev: TCCGACCTTAGAATCTGCTACTT |
| *Pdzk1* | Fwd: TGGAGAAAATGTAGAGAACGCC  Rev: GGAGGAACATGATACGGCTTC |
| *Urat1* | Fwd: GGAGGAACCAAGCAGGGACAAA  Rev: CCGTAGAAGGTGAAGCCAAAGG |
| *Slc17a3* | Fwd: GGGCCTAAGCCAGTCCTCAA  Rev: GAAGACAAAGGGCCACCCAA |
| *Il-10* | Fwd: GCCCAGAAATCAAGGAGCATT  Rev: TTGGAGCTTATTAAAATCACT |
| *Arg-1* | Fwd: AGCACTGAGGAAAGCTGGTC  Rev: CAGACCGTGGGTTCTTCACA |
| *CD206* | Fwd: TAGCACTGGGTTGCATTGGT  Rev: GCAGTGTTAAGACGCTAGTGC |
| *Tnf-α* | Fwd: GATCGGTCCCCAAAGGGATG  Rev: GTGGTTTGTGAGTGTGAGGGT |
| *Xo* | Fwd:ATGGCAAAAAGGTGGTGGAGA  Rev:GCAACATGATGCAAGGAGCA |
