# Supplementary material for: Bergenin as a Novel Urate-Lowering Therapeutic Strategy for Hyperuricemia
Source: Front Cell Dev Biol. 2020 Jul 29;8:703. doi: 10.3389/fcell.2020.00703 (PMC7403512; doi:10.3389/fcell.2020.00703)
Supplement: Supplementary file 6 [file Presentation_1.pdf]

Supplementary Figure S1: (A) The mRNA expression of xanthine oxidase in kidneys and intestine. (B) The xanthine oxidase activity in ileum and jejunum.

Supplementary Figure S2: Histopathological analysis of intestine tissues after H&E staining.

Supplementary Figure S3: Representative immunofluorescence images showing ABCG2 expression (red) and SLC2A9 expression (green) in colon, ileum and jejunum. Nuclei were stained with DAPI (blue).

Supplementary Figure S4: HK-2 and Caco-2 cells were transfected with *SIRT1* siRNA or scrambled siRNA for 48 h. Cells were then pretreated with or without bergenin for 2 h, followed by incubation with 8 mg/dL UA for another 10 h. Representative western blots showing ABCG2, SLC2A9, SIRT1 and PPARγ protein levels.
